# Plasma Membrane-Located Purine Nucleotide Transport Proteins Are Key Components for Host Exploitation by Microsporidian Intracellular Parasites

**DOI:** 10.1371/journal.ppat.1004547

**Published:** 2014-12-04

**Authors:** Eva Heinz, Christian Hacker, Paul Dean, John Mifsud, Alina V. Goldberg, Tom A. Williams, Sirintra Nakjang, Alison Gregory, Robert P. Hirt, John M. Lucocq, Edmund R. S. Kunji, T. Martin Embley

**Affiliations:** 1 Institute for Cell and Molecular Biosciences, The Medical School, Newcastle University, Newcastle upon Tyne, United Kingdom; 2 Department of Microbiology, Monash University, Melbourne, Victoria, Australia; 3 Victoria Bioinformatics Consortium, Monash University, Melbourne, Victoria, Australia; 4 School of Medicine, University of St. Andrews, St. Andrews, United Kingdom; 5 Medical Research Council, Mitochondrial Biology Unit, Cambridge, United Kingdom; University of Virginia Health System, United States of America

## Abstract

Microsporidia are obligate intracellular parasites of most animal groups including humans, but despite their significant economic and medical importance there are major gaps in our understanding of how they exploit infected host cells. We have investigated the evolution, cellular locations and substrate specificities of a family of nucleotide transport (NTT) proteins from *Trachipleistophora hominis*, a microsporidian isolated from an HIV/AIDS patient. Transport proteins are critical to microsporidian success because they compensate for the dramatic loss of metabolic pathways that is a hallmark of the group. Our data demonstrate that the use of plasma membrane-located nucleotide transport proteins (NTT) is a key strategy adopted by microsporidians to exploit host cells. Acquisition of an ancestral transporter gene at the base of the microsporidian radiation was followed by lineage-specific events of gene duplication, which in the case of *T. hominis* has generated four paralogous NTT transporters. All four *T. hominis* NTT proteins are located predominantly to the plasma membrane of replicating intracellular cells where they can mediate transport at the host-parasite interface. In contrast to published data for *Encephalitozoon cuniculi*, we found no evidence for the location for any of the *T. hominis* NTT transporters to its minimal mitochondria (mitosomes), consistent with lineage-specific differences in transporter and mitosome evolution. All of the *T. hominis* NTTs transported radiolabelled purine nucleotides (ATP, ADP, GTP and GDP) when expressed in *Escherichia coli*, but did not transport radiolabelled pyrimidine nucleotides. Genome analysis suggests that imported purine nucleotides could be used by *T. hominis* to make all of the critical purine-based building-blocks for DNA and RNA biosynthesis during parasite intracellular replication, as well as providing essential energy for parasite cellular metabolism and protein synthesis.

## Author Summary

Microsporidians are highly reduced obligate intracellular eukaryotic parasites that cause significant disease in humans, animals and commercially relevant insects. Despite their medical and economic interest the mechanisms whereby microsporidians exploit the cells they infect are mainly unknown. We have characterised a conserved family of nucleotide transport proteins that we demonstrate have key roles in parasite biology. Microsporidians cannot synthesize the primary building blocks needed to make DNA and RNA for themselves, so they must import the starting materials from the infected host. We show that the microsporidian *Trachipleistophora hominis*, originally isolated from an HIV/AIDS patient, may achieve this by using four nucleotide transport proteins located in the plasma membrane of replicating intracellular parasites. In functional assays we demonstrate that all four proteins can transport radiolabelled adenine and guanine nucleotides. Genome analysis suggests that the imported nucleotides could be transformed by *T. hominis* into all of the critical purine-based building-blocks needed for DNA and RNA biosynthesis during parasite intracellular replication, as well as providing essential energy for parasite cellular metabolism and protein synthesis.

## Introduction

Microsporidian parasites are highly reduced eukaryotes that have an obligate intracellular lifestyle based upon the exploitation of other eukaryotic cells [1]. The life cycle of microsporidians alternates between a dispersive spore stage that is resistant to environmental stress, and intracellular replicative stages that can only take place inside the cytoplasm of an infected host cell. Despite lineage-specific variations [1], the general infectious cycle starts with spore germination and the injection of the parasite through a specialised polar tube into the cytoplasm of a suitable host cell. The active vegetative cell (meront) then undergoes binary fission, and after several rounds of multiplication, differentiates (sporogony) into spores that can exit the host by either cell lysis or exocytosis to infect adjacent cells and tissues or a new host [2], [3]. Microsporidians are a large group of parasites with over 1200 described species infecting most animal groups including economically important fish, insect pollinators and silkworms [1], [2], [4], [5]. Microsporidians are also increasingly recognised as a significant threat to human health, affecting not only patients with HIV/AIDS but also the young and old in the developing world [6].

A hallmark feature shared by microsporidians and bacterial obligate intracellular pathogens is a dramatic genomic reduction coupled with loss of metabolic pathways during the transition from a free-living to an obligate intracellular lifestyle [7]. Genome analyses suggest that all microsporidians have lost the tricarboxylic acid (TCA) cycle and oxidative phosphorylation pathways for ATP production although, with a single exception [5], [8], they have retained the pathway for glycolysis [2], [7], [9], [10], [11]. Published data for *Nosema grylli* and *Trachipleistophora hominis* suggest that glycolysis may be mainly active in the spore stage [10], [12] and hence actively replicating parasites living inside host cells may require an alternative source of ATP. In the case of *Encephalitozoon cuniculi* this energy gap is potentially filled by the expression of nucleotide transport (NTT) proteins on the parasite cell surface, where they can be used to import ATP from the host cytoplasm [7], [13]. The same type of transport proteins are also used by important, phylogenetically diverse bacterial intracellular pathogens, including *Rickettsia* and *Chlamydia*, to import host-generated ATP to support their own reduced metabolism [14], [15], [16], [17]. The broad taxonomic distribution of NTT proteins suggests that intracellular pathogens are using lateral gene transfer to exchange transporter genes [13], [18], providing a general strategy for exploiting host cells. Genes for NTT-like transport proteins have been identified in all microsporidian genomes and were recently identified in the genome of the fungal endoparasite *Rozella allomyces* [11], [19]. Phylogenomic analyses demonstrate that *Rozella* and microsporidia share a common ancestor, confirming microsporidia as fungi and suggesting [11], [19] that the acquisition of NTT transporters was a key step for the foundation of a major clade of endoparasitic fungi.

In addition to the loss of mitochondrial ATP-generating pathways, the microsporidians studied so far also lack the enzymes needed for the *de novo* synthesis of the building blocks of DNA and RNA [7]. Loss of the early steps of purine and pyrimidine biosynthesis, which are costly in terms of ATP, has occurred repeatedly among parasitic protozoa, which have devised a variety of ways of securing and interconverting purines and pyrimidines of host origin [20]. Intracellular bacteria also show loss of pathways for *de novo* synthesis of purines and pyrimidines. These bacterial pathogens use their NTT proteins to import a range of different nucleotides in addition to ATP, including GTP, UTP and CTP. In the case of *Protochlamydia amoebophila*, a bacterial symbiont of the protozoan *Acanthamoeba*, these substrates appear to provide all of the starting materials needed to make DNA and RNA [14], [15]. Competition studies on the four NTT transporters of *E. cuniculi* expressed in *Escherichia coli* indicate that ATP transport is reduced by an excess of some nucleotides, but the actual transport of substrates other than ATP and ADP was not directly investigated [13]. In addition to using its NTT transporters to exploit its host, *E. cuniculi* also targets an NTT transporter to its highly reduced mitochondrion (mitosome) to provide ATP for an organelle that can no longer make its own [13]. Like the other microsporidians for which genome sequences are available [11], *E. cuniculi* and *T. hominis* have lost all genes for members of the mitochondrial carrier family of proteins [7], [10]: one member of this family is used by canonical mitochondria to transport ATP and ADP [21]. The mitosomes of *E. cuniculi* contain mitochondrial heat shock protein Hsp70, which requires ATP for its functions in protein import [22] and Fe/S cluster biosynthesis [23], [24]. Other microsporidians, including *T. hominis* [25], also contain ATP-requiring mitochondrial Hsp70 proteins in their mitosomes, but it is not known if the organelles of these species use NTT transport proteins to import ATP.

In the present study we have investigated the evolution, cellular locations and substrate specificities of the nucleotide transport (NTT) proteins of *T. hominis* [10], a microsporidian that is distantly related to *E. cuniculi* [26]. Our results demonstrate that the use of surface-located NTT transport proteins is a general strategy adopted by microsporidians to exploit host cells. Acquisition of an ancestral transporter gene at the base of the microsporidian radiation was followed by lineage-specific events of gene duplication, which in the case of *T. hominis* has generated four paralogous NTT transporters. All four *T. hominis* NTT proteins are located predominantly to the plasma membrane of replicating parasites. In contrast to *E. cuniculi*, we found no evidence for a mitosomal location for any of the *T. hominis* NTT transporters, consistent with lineage-specific differences in transporter and mitosome evolution. All of the *T. hominis* proteins transported purine nucleotides when expressed in *E. coli*, but did not transport pyrimidine nucleotides. Analysis of the enzyme repertoire predicted from the *T. hominis* genome suggests that imported purine nucleotides could be transformed into all of the critical purine-based building-blocks required for DNA and RNA biosynthesis as well as providing essential energy for cellular metabolism and protein synthesis by replicating intracellular parasites.

## Results/Discussion

### *Trachipleistophora hominis* and other microsporidians cannot make nucleotides *de novo* but retain a suite of enzymes for their interconversions

**Figure 1:**
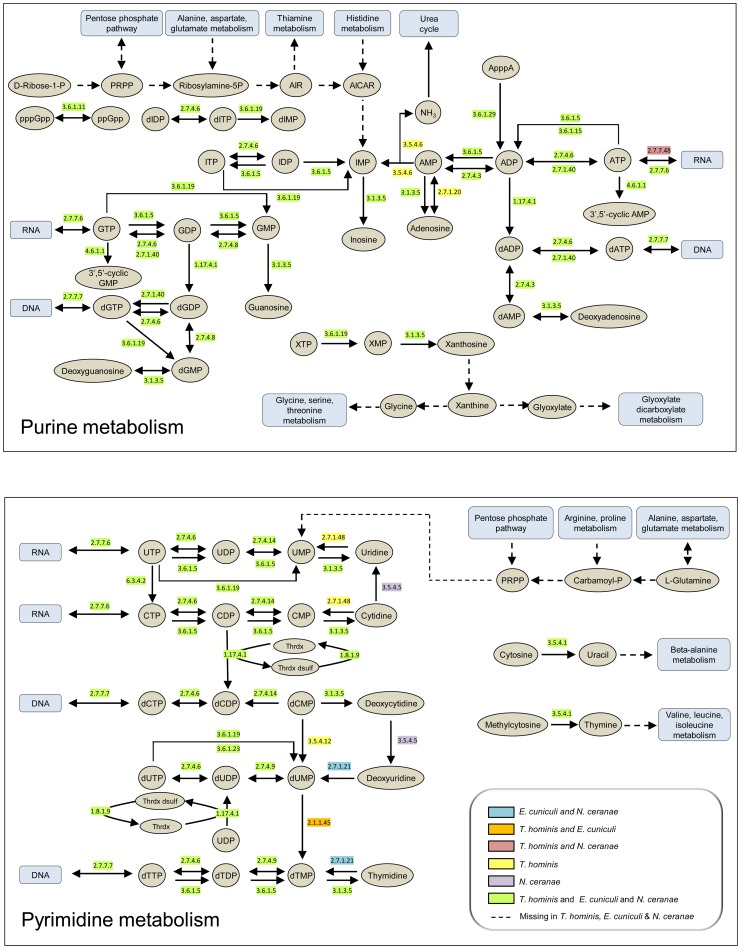
Purine and pyrimidine metabolism in *Encepthalitozoon cuniculi, Nosema ceranae* and *Trachipleistophora hominis*. Enzymes predicted from analysis of published genomes [7], [10], [52] to be present in all three microsporidians are highlighted in green and those for which the genes were detected only in *T. hominis* in yellow. Other colours are as indicated in the key. Dashed lines with arrows indicate enzymes or pathways that appear to be entirely or partly missing in the genome sequences of all three microsporidians [7], [10], [52]. A list of enzymes and EC numbers for the microsporidians and representative intracellular bacteria is given in Table S1. The metabolic schemes for purine and pyrimidine biosynthesis were adapted from KEGG [27].

Nucleotides are the building blocks of DNA and RNA, and also play key roles as signalling molecules and carriers of energy and electrons. They can be made by *de novo* synthesis pathways in free-living Bacteria, Archaea and eukaryotes [27]. In contrast, the loss of the ability to synthesize nucleotides *de novo* appears to be a general feature of microsporidia [11], including *T. hominis* (Fig. 1, Table S1), that is shared with obligate intracellular bacteria such as *Chlamydiae* and *Rickettsiae* [15], [28]. Comparing the manually-curated enzyme complements of *T. hominis*, *E. cuniculi* and *Nosema ceranae* with representative intracellular bacteria (Fig. 1, Table S1) identified a similar core of enzymes for the transformation of purine and pyrimidine nucleotides between different phosphorylation and oxidation states to meet different metabolic requirements. There are minor differences between microsporidians in the enzymes detected by genome analyses (Fig. 1, Table S1), which may reflect differences in the range of substrates that can be used by individual microsporidians. For example, *T. hominis* is predicted to possess a dCMP deaminase (EC 3.5.4.12) potentially capable of converting dCMP into dUMP, that appears to be missing from the genomes of *E. cuniculi* and *N. ceranae* (Fig. 1, Table S1). *T. hominis* also has a gene for uridine kinase (EC 2.7.1.48) which can potentially convert uridine plus ATP into UMP and ADP, that is missing from *E. cuniculi* and *N. ceranae* (Fig. 1, Table S1). We did not detect any *T. hominis*, *E. cuniculi* or *N. ceranae* enzymes or pathways that could potentially convert between adenine and guanine nucleotides, or between purine and pyrimidine (cytosine, uracil and thymidine) nucleotides. This suggests that *T. hominis* and other microsporidians need to import both types of purine nucleotides and at least one type of pyrimidine nucleotide, or substrates that can be used to make them, to complete DNA and RNA biosynthesis during intracellular replication.

### The genome of *Trachipleistophora hominis* contains genes for four putative nucleotide transport (NTT) proteins

The *T. hominis* genome contains genes for four nucleotide transport (NTT) proteins [10]. All four proteins are predicted to contain secondary structure elements typical of characterised NTT proteins [29], including 11 to 12 predicted alpha-helical transmembrane domains (TMDs) (Figure S1) and associated intracellular and extracellular loop regions. Based upon published data it is not possible to predict the range of substrates that can be transported by any particular NTT directly from primary sequence comparisons, although all four *E. cuniculi* proteins and most of the NTT proteins characterised to date for bacteria are able to transport ATP [13], [15], [17], [28], [30], [31]. Four charged residues (K155, E245, E385 and K527 [31]) are strongly conserved among bacteria, in *Rozella allomyces*, in most published microsporidian sequences including all four *E. cuniculi* NTT sequences, and in *T. hominis* ThNTT2 (uniprot L7JXU1) and ThNTT4 (uniprot L7JS26) (Figure S1). All four residues were previously shown to be important for the transport mechanism of the *Arabidopsis* plastid ADP/ATP transporter AATP1 [31] and mutation of K527 also reduced P_i_ transport by the *Protochlamydia amoebophila* ADP/ATP transporter NTT1 (residue K446 in the *P. amoebophila* sequence [30]). The predicted amino acid sequences of ThNTT1 (uniprot L7JRV4; I155, N245, Y385) and ThNTT3 (uniprot L7JTX7; I155, V245 and Y385) have non-conservative changes at three of the four alignment positions, but there are conserved amino acids of the correct identity within 3 or 4 residues in both sequences (Figure S1). Based upon published information for *Arabidopsis* [31] and *Protochlamydia* [15] the conserved lysine (K527) is thought to be important for the transport of nucleoside triphosphates, but not for transport of nucleoside diphosphates.

**Figure 2:**
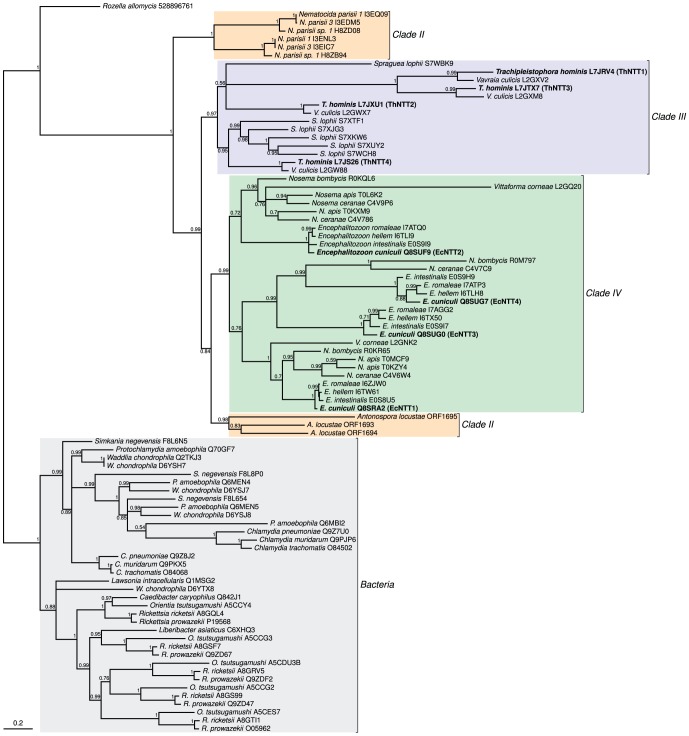
The evolution of microsporidian nucleotide transport proteins is characterized by an initial horizontal acquisition of a single gene followed by lineage-specific gene duplications. A phylogeny of NTT proteins for the microsporidian/*Rozella* clade of endoparasitic fungi and representative bacterial intracellular pathogens including *Chlamydia* and *Rickettsia*. The tree suggests a single common origin for NTT proteins in *Rozella* and microsporidians followed by the lineage- and clade-specific gene duplications that define microsporidian NTT protein evolution. The different clades of microsporidians were defined previously [26]. A similar pattern of repeated gene duplication is evident in the tree for bacterial proteins. Values at nodes are Bayesian posterior probabilities.

To investigate the evolution of the *T. hominis* proteins relative to those from other microsporidians, *R. allomyces*, and bacterial outgroups, we carried out a detailed phylogenetic analysis (Fig. 2). The common endoparasitic ancestor of *R. allomyces* and microsporidia is most parsimoniously inferred to have had a single NTT gene [19]. Based upon the absence of any deep symmetrical split in the tree of microsporidian NTTs (Fig. 2), it appears likely that the common ancestor of microsporidians also possessed a single NTT gene. The variable number of NTT genes detected in the contemporary microsporidian genomes investigated appears to be the product of repeated events of lineage-specific gene duplication. Hence the common ancestor of *T. hominis* and *Vavraia culicis* probably had four paralogous NTT genes; by contrast, their close relative *Spraguea lophii* has six NTT genes [32]. The common ancestor of the three *Nematocida* isolates had only two genes for NTT transport proteins. The common ancestor of *Encephalitozoon* and *Nosema* species probably had four NTT genes, one of which was subsequently lost by *Nosema* spp.

According to classical theory [33] gene duplication can have a number of potential advantages. For example, an increased gene dosage effect could increase the amount of NTT protein produced, or relaxed selection on individual gene copies might allow functional divergence of NTT proteins in terms of their substrate specificities, expression patterns or cellular location. It is interesting to note that *T. hominis* ThNTT4 is more conserved than the other *T. hominis* paralogues (Fig. 2, Figure S1), and groups closely with related NTT sequences from *V. culicis* and *S. lophii*; one possibility is that ThNTT4 carries out the ancestral NTT function for the clade [34]. This protein was also the only ThNTT homologue detected in a recent investigation of the spore proteome of *T. hominis* [10]. Two of the other *T. hominis* NTT genes (ThNTT1 and ThNTT3) and their respective *V. culicis* orthologues are more divergent and have lost or shifted the position of broadly-conserved residues (Figure S1) previously implicated in transport function [31], suggesting relaxed selection and possibly functional divergence within the *T. hominis*/*V. culicis* clade. Bacterial NTT proteins have been studied in greater detail than those of microsporidians and there is evidence that gene duplication events have allowed functional divergence in the transport mechanism and substrate specificities of individual proteins [14], [15]. The published functional data for the four *E. cuniculi* NTT proteins demonstrates that they can all transport ATP when expressed in *E. coli* and, although other substrates have not been directly evaluated for transport, competition experiments with different nucleotides yielded broadly similar inhibition profiles for all four *E. cuniculi* NTT proteins [13].

The most compelling evidence for functional specialisation affecting *E. cuniculi* NTT proteins comes from their different cellular locations. Three of the *E. cuniculi* NTT proteins are located on the surface of the parasite but the fourth (*E. cuniculi* EcNTT3) is targeted to its mitosomes. Orthologues of *E. cuniculi* EcNTT3 were also found in *E. romaleae*, *E. hellem* and *E. intestinalis*, but were not detected in *Nosema* species or other microsporidians. Note that it is unclear whether the cellular localizations and transport specificities of these genes can be transferred to other microsporidian NTTs, because the gene duplications giving rise to the four *E. cuniculi* NTTs do not date back to the last common ancestor of microsporidia. In particular, the observation that the other three *E. cuniculi* NTT paralogues are surface-located, a feature shared with bacterial NTT homologues [15], suggests that the targeting of *E. cuniculi* EcNTT3 to the mitosome is a derived state that might be restricted to the *Encephalitozoon* lineage. Computational analyses detected no obvious differences between *E. cuniculi* EcNTT3 and surface-located *E. cuniculi* NTT paralogues that might explain the observed differential targeting [13], and genetic manipulation of microsporidians to identify the specific residues involved is still not possible. Mitosomal targeting in general is not well understood in microsporidia, and even for model mitochondria the precise targeting signals are known for only a subset of organelle proteins [35], [36]. In order to investigate the locations of the four *T. hominis* NTT proteins we therefore made specific antibodies and carried out detailed immuno-localisation experiments.

### All four *T. hominis* NTTs are located in the plasma membrane at the host-parasite interface

**Figure 3:**
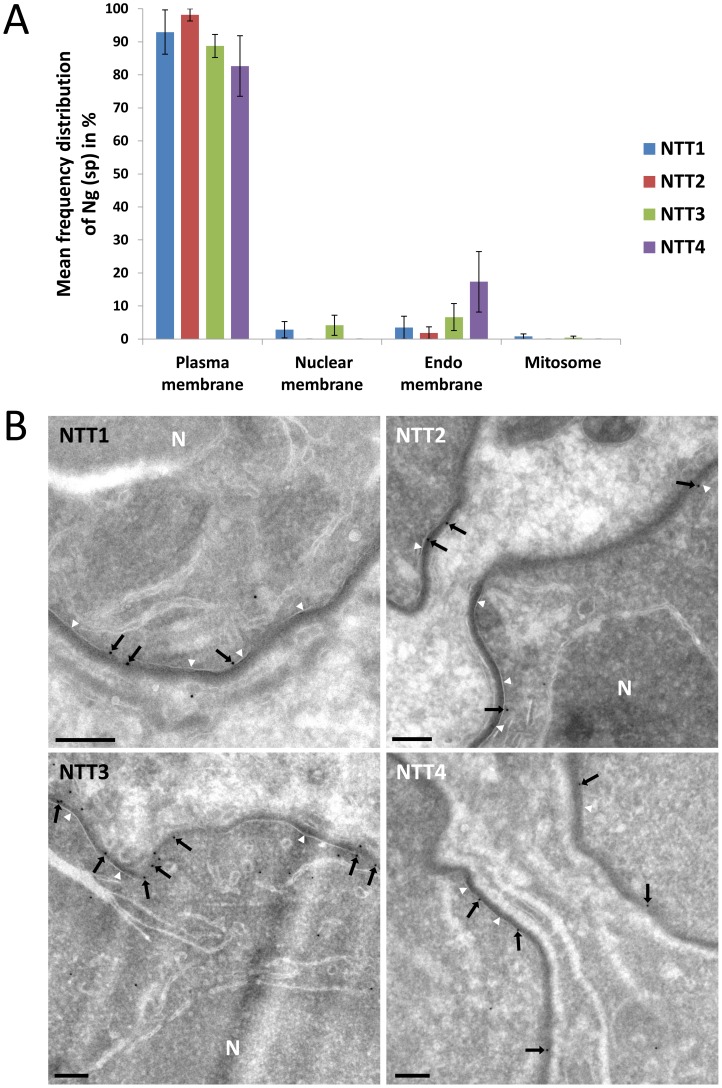
Localisation of individual ThNTT using quantitative immuno-electron microscopy. (A) Frequency distribution of specific gold label (Ng(sp)) for all four ThNTTs (mean of 3 individual experimental values). The labelling density was assessed by estimating the membrane profile length of compartments of interest using intersection counting [37], [53] and counts of membrane-associated gold particles. The labelling density for each ThNTT antibody was compared with the corresponding labelling density obtained in a peptide or polypeptide-inhibition control. The distribution of specific gold label was then calculated by multiplying the fraction of specific labelling over each compartment with the initial gold particle counts. Error bars represent standard errors of the mean. (B) Thawed cryo-sections of *T. hominis*-infected RK cells labelled with antisera against the four ThNTTs. Micrographs are representative of the quantitative data presented above. Membrane-associated gold particles (black arrows) demonstrate the localisation of all four ThNTTs at the plasma membrane (white arrowheads) of the parasite. The plasma membrane is visible as a smooth membrane profile covered with an electron dense coating. Partial profiles of cells in meront stages are shown. N = nucleus; bars = 200 nm.

The intracellular localisation of the four *T. hominis* ThNTTs was analysed using quantitative immuno-electron microscopy. Thawed cryo-sections of *T. hominis*-infected RK cells were labelled with antisera raised against each of the four ThNTTs and the gold-label quantified using methods that ensure precise and unbiased quantification [37]. The specificity of each ThNTT was determined quantitatively in vegetative stages (meronts) by assessing the extent to which the specific peptides (for ThNTT1, 2 and 3) or polypeptide (for ThNTT4) that were used to generate the antisera blocked the individual antibody signals in parallel replicate experiments [38], [39] (see Material and Methods and Figure S2). The predominant localisation of all four ThNTTs was in the plasma membrane of the parasite (Fig. 3, Figure S2). Specific labelling was virtually absent over the mitosome but was detectable within intracellular membranes (for ThNTT3 and 4), which were mainly composed of tubulovesicular and cisternal profiles. These compartments may comprise elements of the Golgi complex and endoplasmic reticulum, but the absence of compartment-specific markers for these studies make their exact assignment problematic at present. Nevertheless, it appears possible that the specific signal over these internal membranes represents ThNTTs in transit through the secretory pathway.

**Figure 4:**
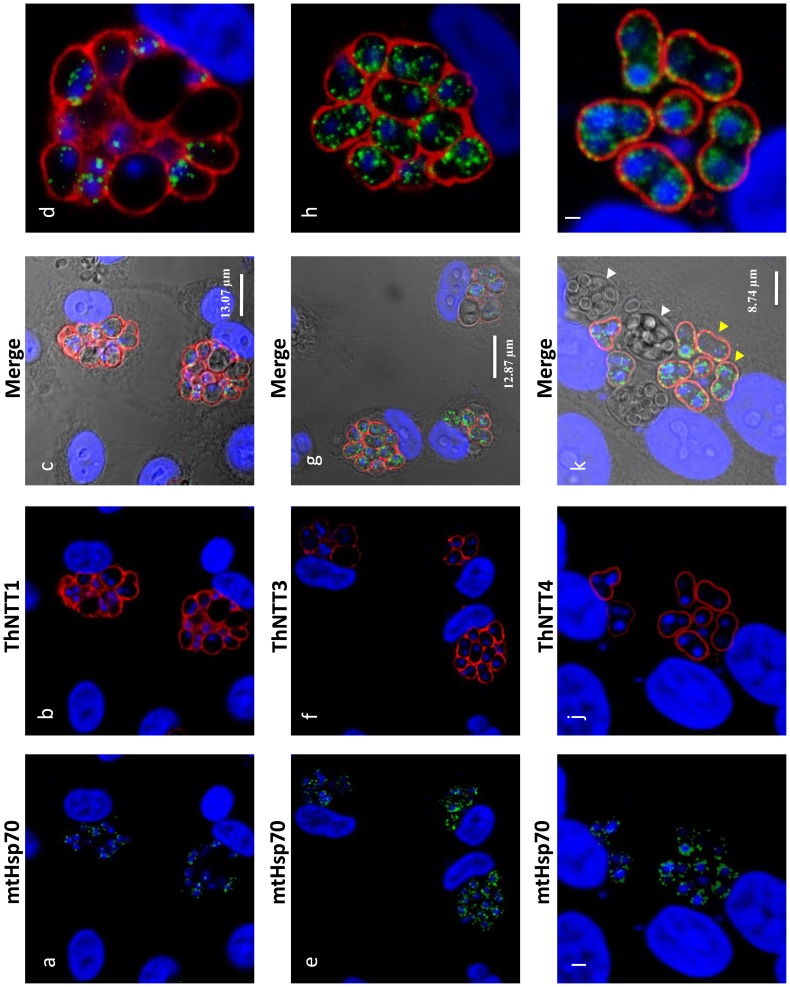
Plasma membrane localisation of ThNTT1, ThNTT3 and ThNTT4 demonstrated by fluorescence microscopy. Rat antibody to the mitosomal marker Hsp70 (**a**, **e** and **i**) labelled discrete structures (mitosomes) inside the parasites (green) whereas rabbit antisera to ThNTT1 (**b**), ThNTT3 (**f**) and ThNTT4 (**j**) labelled the surface of the parasite (red). The nuclei of rabbit kidney host cells (large nuclei) and parasites (small nuclei) were labelled with DAPI (blue). **c, g** and **k**; DIC images with the three merge channels. **d**, **h** and **l**, enlarged images of individual clusters of *T. hominis* showing antibody labelling. White arrow shows aggregation of spores within the host cell. Yellow arrows show the meront or vegetative stage of the parasite.

The antibodies were also used on *T. hominis*-infected rabbit kidney cells prepared for immunofluorescence analysis (IFA) to gain an overview of the ThNTT distribution in replicating parasites. The distribution of staining for antibodies raised for ThNTT1, ThNTT3 and ThNTT4 demonstrate a localisation on the surface of the growing parasites (Fig. 4), consistent with the plasma membrane location revealed by the EM data. As illustrated in Fig. 4k, the antibodies against ThNTT4 and ThHsp70 did not give signals for structures inside the developing thick walled spores contained in the sporophorous vesicles (SPV) that are a characteristic feature of the *T. hominis* intracellular lifecycle [40], [41]. We suspect that this is due to a lack of permeability of the developing spore wall to antibodies because both proteins are detected in spore digests analysed using proteomic methods [10]. The lack of label in the outer envelope of the SPV is consistent with our EM data where no signal was detected for ThNTT4 in the electron-dense outer layer surrounding the parasites (Fig. 3) from which the SPV envelope is thought to originate [41]. We were unable to detect any specific staining in IFA of parasites using the antibody against ThNTT2 despite employing different fixation procedures and making a second polyclonal antibody against segments of several predicted extracellular loops of ThNTT2 as previously described [13] (Table S3 in Text S1). Our failure to obtain IFA data for ThNTT2 despite successful EM experiments for this protein may reflect differences in sample preparation influencing epitope accessibility: the immuno-EM approach includes opening up the compartments by sectioning whereas IFA involves permeabilization of cell membranes and depends on penetration of the antibody prior to labelling.

In summary, we could localise all *T. hominis* NTTs to the plasma membrane of vegetative cell stages that are growing and replicating inside the host. In contrast to published data for *E. cuniculi* [13], there was no evidence for a mitosomal localisation of any of the four *T. hominis* NTTs. It has been demonstrated that the mitosomes of *E. cuniculi* and *T. hominis* contain proteins of the essential Fe/S cluster biosynthesis pathway [23], [24], [25], which in model organisms requires ATP for several steps [24]. It is possible that the mitosomes of *T. hominis* use other transport proteins to import ATP, but candidates for this role are difficult to predict solely from genome analyses [10] and there is as yet no proteomics data for *T. hominis* mitosomes. In classical mitochondria, the transport of metabolites across the inner membrane is highly selective in order to maintain the electrochemical proton gradient used for ATP synthesis. Since the mitosomes of *T. hominis* no longer make ATP it is also possible that selection for an impermeable inner membrane has been sufficiently relaxed to allow passive transport of ATP through the inner membrane translocase (TIM) channel [10] of the mitosome protein import pathway. Interestingly, a similar conundrum exists for ATP supply to the mitosome of the extracellular parasite *Giardia lamblia* [42]. The genome of this parasite lacks genes for mitochondrial ATP generation and mitochondrial carrier family proteins, and it also lacks genes for NTT proteins [43]. Nevertheless, its mitosomes can still make Fe/S clusters [44], [45] suggesting that ATP is available to support this pathway inside the organelle.

### *Trachipleistophora hominis* NTTs transport adenine and guanine nucleotides, but not pyrimidine nucleotides, when expressed in *E. coli*

**Figure 5:**
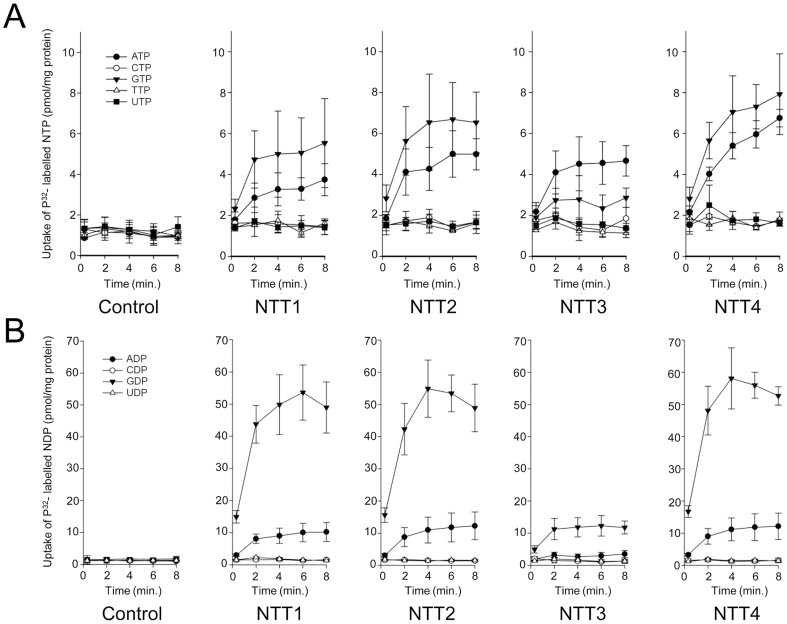
All four NTT proteins of *Trachipleistophora hominis* transport purine nucleotides but not pyrimidine nucleotides. Uptake of [α-32P]-labelled (A) nucleoside triphosphates or (B) nucleoside diphosphates into IPTG-induced *E. coli* cells containing a pET16b vector encoding the respective ThNTT genes or no insert (control). For all nine nucleotides the data are represented by the mean and standard error of two independent experiments.

To identify the transported substrates of the four *T. hominis* NTTs we expressed the proteins in *Escherichia coli* and carried out transport experiments in whole cells [13] with nine different ^32^P-labeled nucleotides. ThNTT1-4 transported ATP and GTP over background levels measured for *E. coli* containing the vector only (Fig. 5A). ThNTT1, 2 and 4 transported GTP with higher rates than ATP, but the differences were not statistically significant; by contrast, ThNTT3 transported ATP slightly faster than GTP (Fig. 5A). The import of ATP and GTP by ThNTTs expressed in the plasma membrane of intracellular *T. hominis* could provide purine-based substrates for DNA and RNA biosynthesis as well as energy for protein synthesis during parasite replication. ^32^P-labeled CTP, TTP and UTP were not taken up as the accumulation levels were similar to the *E. coli* vector-only control. Uptake experiments using radiolabelled ^32^P-labeled nucleoside diphosphates demonstrated a significant preference for transport of GDP over ADP for all four ThNTTs expressed in *E. coli*. Import of ADP and GDP would provide substrates for the ATP-activated ribonucleoside diphosphate reductase (EC 1.17.4.1) that provides an essential link between parasite RNA and DNA biosynthesis (Fig. 1, Table S1). Based upon our analysis of its genome (Fig. 1, Table S1), *T. hominis* should be able to synthesise all of the purine-based components of DNA and RNA given the import of both adenine and guanine nucleotides. Our analyses suggest that *E. cuniculi*, *N. ceranae* (Fig. 1, Table S1) and potentially other microsporidians will have a similar requirement and capacity. Accumulation of ^32^P^−^labeled CDP or UDP in the NTT expressing strains was similar to the *E. coli* control, showing that these pyrimidine diphosphates were not transported (Fig. 5B). It is clear that the NTTs of *T. hominis* transport purine nucleotides, but not pyrimidine nucleotides. The apparent absence of genes for enzymes to make pyrimidine nucleotides *de novo* from the *T. hominis, E. cuniculi and N. ceranae* genomes suggests that additional, currently unknown transport processes, are needed to complete DNA and RNA biosynthesis during parasite intracellular replication.

### Conclusions

Microsporidians infect most animal groups, often with devastating consequences for the host animal [1], [2], [5]. Given the major loss of genes affecting most metabolic pathways revealed by genome analyses [7], [11], surface-located transport proteins are of critical importance for completing the microsporidian life cycle once inside an infected host cell. Consistent with this idea, comparative analyses suggest that expansion of specific transporter families was contemporaneous with loss of metabolic capacity at the origin of the microsporidian radiation [10], [11]. We have investigated the evolution, intracellular location and substrate specificities of nucleotide transport (NTT) proteins, homologues of which are conserved on all microsporidian genomes. Gaps in the predicted microsporidian metabolome suggest that these transporters potentially play essential roles supporting microsporidian DNA and RNA metabolism (Fig. 1, Table S1), as well as providing energy for cellular metabolism and protein synthesis for a cellular stage that may no longer make its own [5]. Consistent with predictions from analyses of the *T. hominis* enzyme repertoire that both types of purine nucleotide must be imported, we detected transport of adenine and guanine nucleotides by all four ThNTTs (Fig. 5). Further work is now needed to characterise the detailed mechanisms of transport used by the ThNTTs. It has already been demonstrated that the mitosome-located *E. cuniculi* EcNTT3 is an exchanger of adenine nucleotides exporting ADP in exchange for ATP to supply an organelle unable to make its own ATP [13]. This mechanism (Class I NTT proteins [15]) has already been demonstrated for some NTTs of bacterial intracellular pathogens with a reduced energy metabolism - often called energy parasites - including *Rickettsia prowazekii* and *Chlamydia trachomatis* [15]. The loss of the ability to make mitochondrial ATP and the apparent down-regulation of glycolytic enzymes in replicating cells of *T. hominis* [10] suggests that one or more of the *T. hominis* NTTs might also use this transport mechanism. However, the requirement for net nucleotide import to support DNA and RNA biosynthesis that is predicted by our genome analyses also suggests that at least one of the ThNTTs could mediate a unidirectional proton-energised import of purine nucleotides. This mechanism (Class II [15]) has also been described for NTT transporters from intracellular bacteria that lack *de novo* nucleotide biosynthesis, including *Chlamydia trachomatis* [14] and *Protochlamydia amoebophila* [15]. The similarities in the predicted enzyme repertoires of *E. cuniculi* and *N. ceranae* suggest that the requirement for host produced ATP and net nucleotide import of both types of purine nucleotides may be a general feature of microsporidians. Although NTT-like proteins in some bacteria [15] have been shown to transport purine and pyrimidine nucleotides, we did not detect any transport of the tested pyrimidine nucleotides by the NTTs of *T. hominis* under the assay conditions we used (Fig. 5). It is therefore possible that additional transporters are needed to provide these substrates. One candidate for this function [46] is a conserved family of microsporidian proteins [11] that share significant sequence similarity to *E. coli* NupG [47]. This is a nucleoside transporter of the major facilitator superfamily of transport proteins that can transport both purine (adenosine) and pyrimidine (uridine) nucleosides when expressed in *E. coli* or *Xenopus* oocytes [47]. Genome analyses (Fig. 1, Table S1) suggest that any imported uridine could be used by *T. hominis* to make the pyrimidine nucleotides needed for nucleic acid biosynthesis.

The observed pattern of lineage- and even species-specific duplications of NTTs over the microsporidian tree, coupled with differences in their subcellular localization between *T. hominis* and *E. cuniculi*, suggests that the role of NTTs in parasite biology has continued to evolve throughout the microsporidian radiation. Previous work on the NTT proteins of *Encephalitozoon cuniculi* demonstrated that some NTT proteins were located on the surface of parasites inside infected host cells [13]. Here we demonstrate that all four ThNTTs are located in the plasma membrane of replicating *T. hominis* cells, providing the first detailed evidence for NTT subcellular location. These data suggest that the location of NTT transporters at the host-parasite interface is a general strategy used by microsporidians to exploit host cells and compensate for their own highly reduced metabolism. In contrast to *E. cuniculi* we found no evidence that any of the ThNTTs were located to the *T. hominis* mitosome: this feature appears to be a derived feature of mitosome biology that may be restricted to the *Encephalitozoon* lineage.

## Materials and Methods

### Phylogenetic analyses

All sequences used in this study are provided (Table S2 in Text S1). Sequences were aligned using muscle (v3.8.31, [48]) under the default conditions, and divergent sites were removed using trimAl (v1.2rev59, [49]) with the “-automated1” function. Bayesian phylogenetic trees were inferred with Phylobayes (v3.3e, [50]) under the C20 model (“-catfix C20”) to account for across-site compositional heterogeneity in the data set. Convergence was assessed by using the bpcomp command to monitor the maximum and average discrepancy in bipartitition frequencies between two independent MCMC chains. The analysis was stopped when the maximum difference dropped below 0.1, as recommended by the authors [50]. The sequences, alignment and treefile have been deposited in Figshare (http://dx.doi.org/10.6084/m9.figshare.1104386).

### Organisms and growth conditions

*Trachipleistophora hominis* [40] was grown in RK-13 cells at 37°C in Dulbecco's Modified Eagle Medium (DMEM), containing Kanamycin 100 µg/ml, Penicillin 100 µg/ml, Streptomycin 100 µg/ml, and Fungizone 1 µg/ml. *E. coli* Rosetta 2 (DE3) (Novagen), BL1-AI, C43, pLysS, were grown in LB media (10 g/l tryptone, 5 g/l yeast extract, 5 g/l NaCl, pH 7.5) for routine cloning and expression trials. For uptake studies, *E. coli* Rosetta 2 (DE3) cells were grown in TB media (1.2 g/l peptone, 24 g/l yeast extract, 72 mM K_2_HPO_4_, 17 mM KH_2_PO_4_ and 4 ml/l glycerol). *E. coli* Rosetta 2 (DE3) was routinely grown in media supplemented with 34 µg/ml chloramphenicol, and all strains were grown in media containing 100 µg/ml ampicillin after transformation with the constructs. Cells were grown at 37°C unless indicated otherwise.

### Antibody generation

To obtain antibodies targeting exposed epitopes of these predominantly hydrophobic proteins, we identified two peptide sequences located in predicted surface-exposed loop regions for each ThNTT (Figure S1, Table S3 in Text S1). Peptide synthesis, animal immunisation, antisera extraction and affinity purification of all antisera was performed by Agrisera (Sweden). Both peptides for each ThNTT were used for immunisation of the same rabbit, and the affinity-purified antisera were tested for their specificity against *E. coli* strains expressing the individual ThNTT proteins. The peptide sequences are given in Table S3 in Text S1. The peptide antibodies for ThNTT3 gave some non-specific binding in IFA experiments and antibodies for ThNTT4 gave a high level of nonspecific background labelling in immuno-electron microscopy, so we designed a second set of antibodies to regions of ThNTT3 or ThNTT4 predicted to form exposed loop regions (Figure S1, Table S3 in Text S1). These regions were synthesised (GenScript Inc., USA) as a single gene encoding the polypeptide, cloned into the pQE-40 expression vector (Qiagen) and expressed in *E. coli* M15 [pREP4] cells as single dihydrofolate reductase (DHFR) fusion proteins and processed to make rabbit antibodies (Agrisera, Sweden) as described previously [13].

### Immunofluorescence

Immunofluorescence was performed as described previously [13], [23], and microscopy images were captured using a Leica SP2 confocal microscope.

### Electron microscopy

Monolayer RK cells (RK-13) were infected with *T. hominis* and grown to near confluence. The cells were fixed in 0.5% glutaraldehyde in 0.2 M PIPES buffer (pH 7.2) for 15 min at room temperature, then scraped from the culture dish and pelleted (15 min at 16.000× g). The cells were subsequently washed three times with buffer (5 min per wash) and cryoprotected in 2.3 M sucrose in PBS overnight at 4°C. Small fragments of the cell pellet were mounted onto specimen carriers and plunge-frozen in liquid nitrogen. Eighty nm thick sections were cut at −100°C (EM FC7 ultracryomicrotome; Leica, Vienna, Austria) and mounted on carbon/pioloform-coated EM copper grids (Agar Scientific, Stansted, UK) in drops containing equal volumes of pre-mixed 2.1 M sucrose and 2% w/v methylcellulose. Prior to labelling, grids were washed in ice-cold distilled water (3 times, 5 min each) followed by PBS at room temperature. The sections were then incubated in 0.5% fish skin gelatin (Sigma Aldrich, Poole, UK) in PBS, and labelled using rabbit antisera raised against the four *T. hominis* NTTs, followed by 10 nm protein-A gold (BBI solutions, Cardiff, UK) and contrasted using 2% w/v methylcellulose/3% w/v uranyl acetate (mixed 9∶1). To assess the specificity of labelling, sections were incubated in parallel with antisera which had been pre-mixed with the peptides (for ThNTT1, 2 or 3) or polypeptide (for ThNTT4) used to generate the antibodies, in order to inhibit specific antibody binding (peptide-control). For this purpose, equal volumes of antisera and the corresponding peptides or polypeptide in PBS were mixed for 30 min at room temperature and were then applied to sections in parallel with the native antisera, which were incubated in PBS under the same conditions. For quantification, labelled sections were sampled systematic uniform random (SUR; [51]) in three individual experiments per antibody by taking 16–20 micrographs per sample with a JEOL 1200 EX transmission electron microscope operated at 80 kv using either Ditabis imaging plates (DITABIS Digital Biomedical Imaging Systems AG, Pforzheim, Germany) or a GATAN Orius 200 digital camera (GATAN, Abingdon, Oxon, UK). Mitosomes were sampled separately by comprehensive scanning of all parasites within a randomly selected grid square (22 to 40 micrographs per sample). Tiff files of micrographs were further analysed using Adobe Photoshop CS6. Square lattice grids were randomly placed on each micrograph and used to estimate the length of membrane profiles of compartments of interest by intersection counting (grid spacing either 262, 618 or 914 nm for the plasma membrane and nuclear envelope; 914 nm or 1.54 µm for endo-membranes, including endoplasmic reticulum and Golgi as well as every other non-categorized internal membrane apart from nuclear envelope and mitosome membranes). Gold particles were categorized as being membrane-associated if the particle was less than 1 particle width away from a membrane profile. The specificity of immunogold labelling was assessed as described previously [38], [39]. Briefly, the specific labelling density D(sp) (gold per micron) was estimated by subtracting the labelling density obtained with the peptide-control D(-) from the initial (raw) density D(0). Next, the fraction of the specific labelling F(sp) is given by D(sp)/D(0) and F(sp) is multiplied with the initial labelling counts over each compartment in order to calculate the specific labelling distribution. The vegetative meront stages of *T. hominis* could be identified as single or multinucleated cells proliferating in RK cells. Spore stages were distinguished by the presence of a discernible cell wall and/or the formation of the polar tube. The plasma membrane was visible as a smooth membrane profile covered with an electron dense coating in early meront stages and as a more convoluted membrane profile in later meront stages. Endo-membranes were defined as membrane structures inside the cytoplasm including the endoplasmic reticulum, the Golgi and any other membrane compartment excluding the nuclear envelope and mitosomal membranes. Mitosomes were identified as double membrane bound organelles with minor and major axes measuring between 50 and 300 nm.

### Nucleotide uptake assays in *E. coli* expressing ThNTT transporters

All four full-length *T. hominis* NTT genes were cloned into the expression vector pET16b (Novagen) and the insert was verified by sequencing. NTT2 was inserted between the NdeI and the BamHI sites, and NTT1, NTT3 and NTT4 were inserted between the XhoI and the Bpu1102I sites; the primer sequences are given in Table S4 in Text S1. For uptake experiments, *E. coli* Rosetta 2 (DE3) pLysS cells (Novagen) were transformed with recombinant vectors encoding the ThNTT genes, with the empty pET16b vector used as a control. Cells were grown at 37°C to an OD_600_ of 0.5 in Terrific Broth and transporter expression was induced by the addition of 1 mM isopropyl β-d-1-thiogalactopyranoside (IPTG) following incubation for 16 hours at 18°C. Cells were harvested by centrifugation (6,000 g, 5 min.), washed twice with PBS (8 g/l NaCl, 0.2 g/l KCl, 1.44 g/l Na_2_HPO_4_, 0.24 g/l KH_2_PO_4_, pH 7.4), and resuspended in PBS to a final OD_600_ of 5.0. The cells were kept at 4°C and then pre-incubated for 15 min at 25°C before being used in the uptake assays. Uptake assays with ^32^P-radiolabeled purine and pyrimidine di- and tri-nucleotides (Hartmann) were performed as described previously for ^32^P-ATP uptake [13].

## Supporting Information

Figure S1 **Sequence conservation among representative nucleotide transport proteins.** Sequence alignment of representative NTT protein sequences (Table S2 in Text S1). The alignment shows conserved features of the NTT proteins for *E. cuniculi* and *T. hominis* compared to the transport proteins of selected bacteria. The strength of the green shading is in proportion to the conservation of residues at individual sites, dark green columns being most conserved [54]. Secondary structure predictions using TMHMM [55] for putative transmembrane domains are shown for *T. hominis* NTT1 compared with *Chlamydia trachomatis* NTT1 and *Rickettsia rickettsia* NTT3. The conservation of a 12 transmembrane helix structure is a general feature of these proteins [56].
(PDF)

Figure S2 **Quantitative immuno-electron microscopy reveals subcellular thntt localisation.** Raw labelling density generated by antisera for the four *T. hominis* NTTs (D(0)) and the labelling density obtained after blocking the antisera with the peptides used to raise the antibodies (D(-)) were used to calculate the specific density of gold signal (D(sp)). D(sp) = D(0) - D(-) [38]. The bar chart depicts the mean of 3 individual experiments. Negative values demonstrate the induction of signal over compartments after using the peptide-inhibited antibodies. Error bars represent standard errors of the mean.
(PDF)

Table S1 **Conservation of enzymes for purine and pyrimidine metabolism in yeast,** ***Encephalitozoon cuniculi*** **,** ***Nosema ceranae*** **and** ***Trachipleistophora hominis*** **, and** ***E. coli*** **K12 W3110 and representative intracellular bacteria (** ***Protochlamydia amoebophila*** **UWE25,** ***Chlamydia trachomatis*** **D/UW-3/CX,** ***Rickettsia rickettsii*** **Sheila Smith and** ***Lawsonia intracellularis*** **).**
(XLSX)

Text S1 **Additional supplementary tables S2 to S4. Table S2.** Source and accession numbers for sequences used in the tree diagram (Fig. 2) in main text. **Table S3.** Sequences of the peptides and recombinant proteins used to generate antibodies. **Table S4.** Sequences of primers used in the study.
(XLSX)
