# Supplementary material for: Plasma Membrane-Located Purine Nucleotide Transport Proteins Are Key Components for Host Exploitation by Microsporidian Intracellular Parasites
Source: PLoS Pathog. 2014 Dec 4;10(12):e1004547. doi: 10.1371/journal.ppat.1004547 (PMC4256464; doi:10.1371/journal.ppat.1004547)
Supplement: Figure S1 — Sequence conservation among representative nucleotide transport proteins. Sequence alignment of representative NTT protein sequences (Table S2 in Text S1). The alignment shows conserved features of the NTT proteins for E. cuniculi and T. hominis compared to the transport proteins of selected bacteria. The strength of the green shading is in proportion to the conservation of residues at individual sites, dark green columns being most conserved [54]. Secondary structure predictions using TMHMM [55] for putative transmembrane domains are shown for T. hominis NTT1 compared with Chlamydia trachomatis NTT1 and Rickettsia rickettsia NTT3. The conservation of a 12 transmembrane helix structure is a general feature of these proteins [56]. (PDF) [file ppat.1004547.s001.pdf]

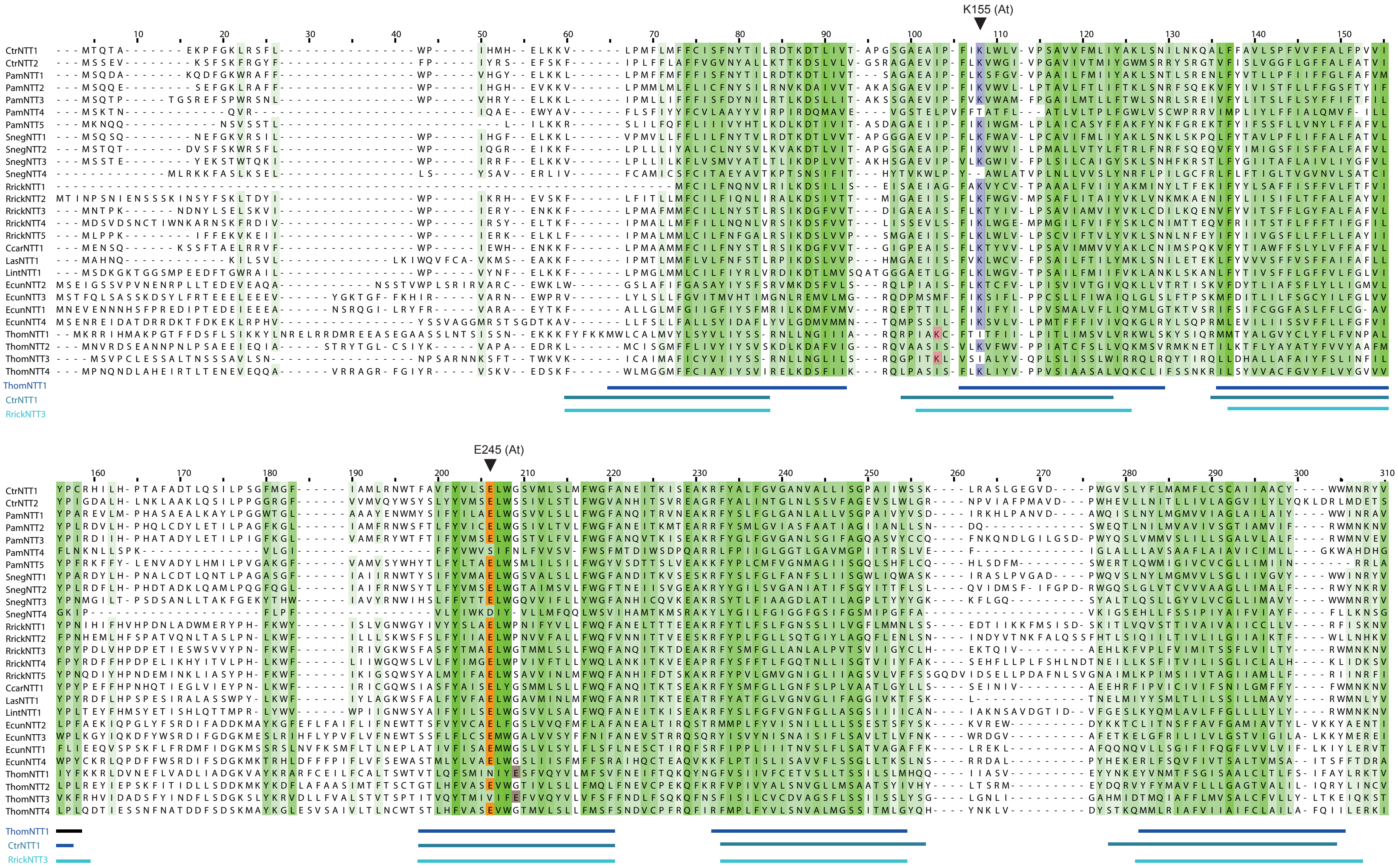

K155 (At)
E245 (At)
CtrNTT1
CtrNTT2
PamNTT1
PamNTT2
PamNTT3
PamNTT4
PamNTT5
SnegNTT1
SnegNTT2
SnegNTT3
SnegNTT4
RrickNTT1
RrickNTT2
RrickNTT3
RrickNTT4
RrickNTT5
CcarNTT1
LasNTT1
LintNTT1
EcunNTT2
EcunNTT3
EcunNTT1
EcunNTT4
ThomNTT1
ThomNTT2
ThomNTT3
ThomNTT4
ThomNTT1
CtrNTT1
RrickNTT3

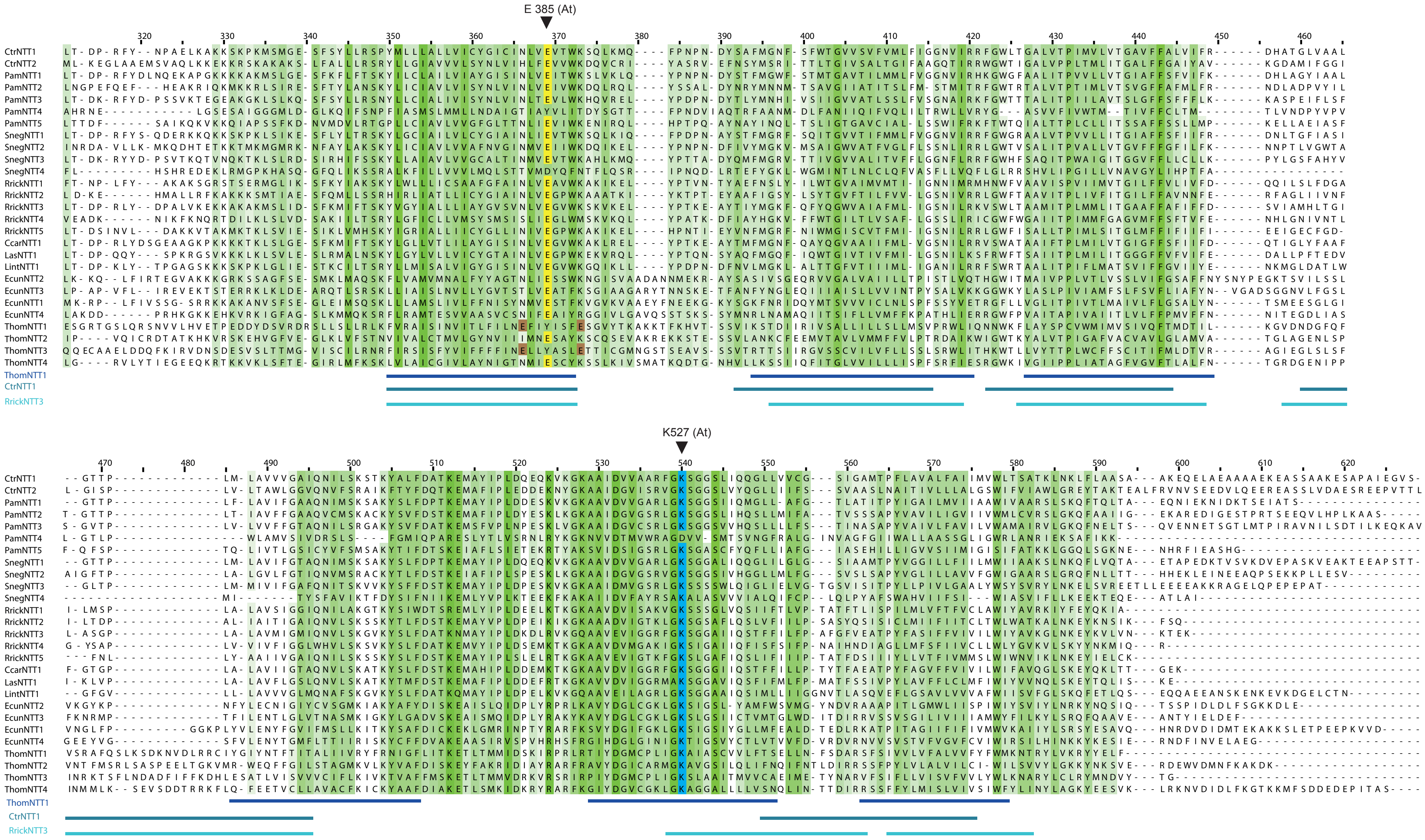

E 385 (At)
320
330
340
350
360
370
380
390
400
410
420
430
440
450
460
CtrNTT1
CtrNTT2
PamNTT1
PamNTT2
PamNTT3
PamNTT4
PamNTT5
SnegNTT1
SnegNTT2
SnegNTT3
SnegNTT4
RrickNTT1
RrickNTT2
RrickNTT3
RrickNTT4
RrickNTT5
CcarNTT1
LasNTT1
LintNTT1
EcunNTT2
EcunNTT3
EcunNTT1
EcunNTT4
ThomNTT1
ThomNTT2
ThomNTT3
ThomNTT4
ThomNTT1
CtrNTT1
RrickNTT3
K527 (At)
470
480
490
500
510
520
530
540
550
560
570
580
590
600
610
620
