# Supplementary figures and images for: Plasma Membrane-Located Purine Nucleotide Transport Proteins Are Key Components for Host Exploitation by Microsporidian Intracellular Parasites
Source: PLoS Pathog. 2014 Dec 4;10(12):e1004547. doi: 10.1371/journal.ppat.1004547 (PMC4256464; doi:10.1371/journal.ppat.1004547)

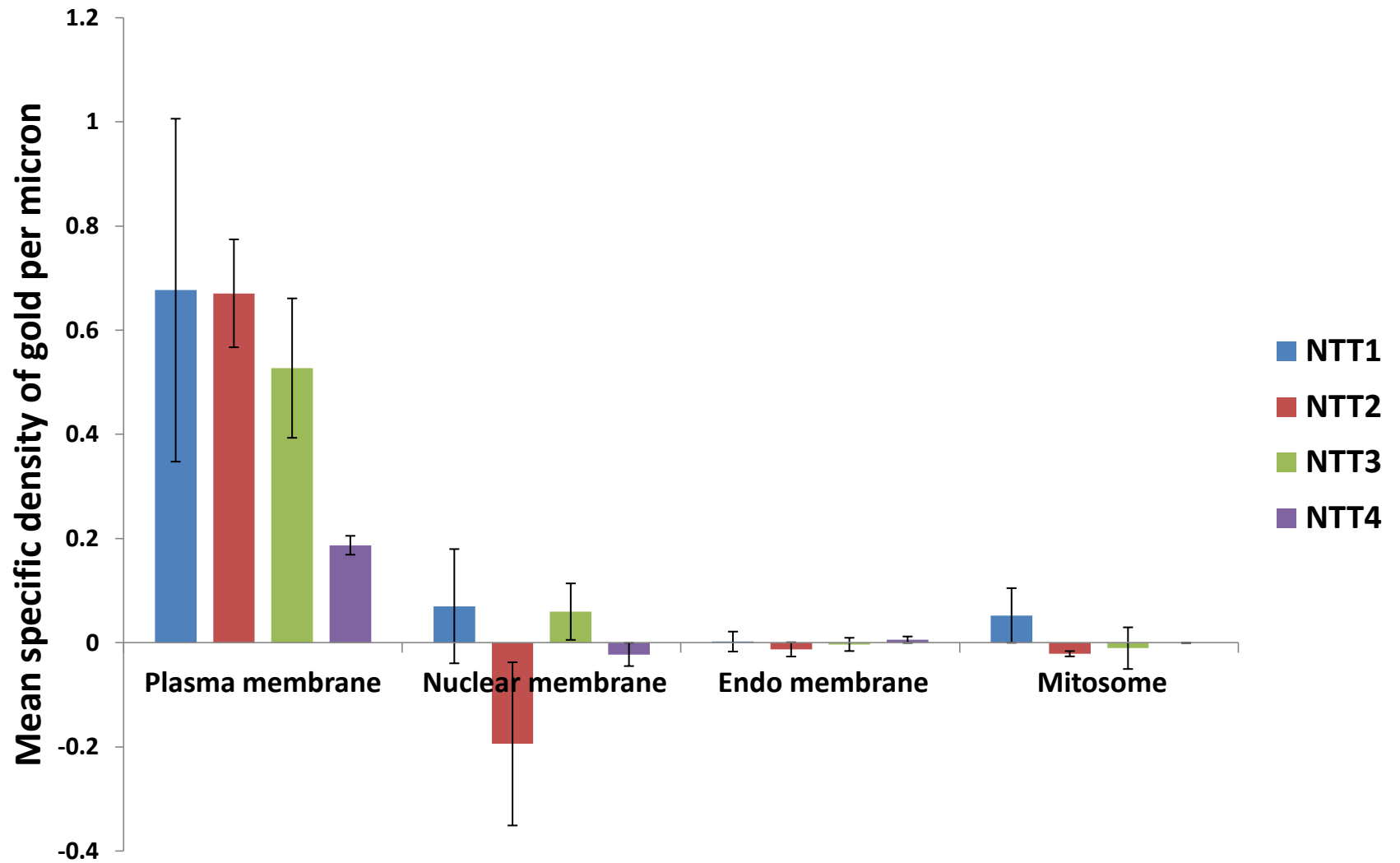

Supplement: Figure S2 — Quantitative immuno-electron microscopy reveals subcellular thntt localisation. Raw labelling density generated by antisera for the four T. hominis NTTs (D(0)) and the labelling density obtained after blocking the antisera with the peptides used to raise the antibodies (D(-)) were used to calculate the specific density of gold signal (D(sp)). D(sp) = D(0) - D(-) [38]. The bar chart depicts the mean of 3 individual experiments. Negative values demonstrate the induction of signal over compartments after using the peptide-inhibited antibodies. Error bars represent standard errors of the mean. (PDF) [file ppat.1004547.s002.pdf]
